# Recent Advances in *Glycyrrhiza glabra* (Licorice)-Containing Herbs Alleviating Radiotherapy- and Chemotherapy-Induced Adverse Reactions in Cancer Treatment

**DOI:** 10.3390/metabo12060535

**Published:** 2022-06-09

**Authors:** Kai-Lee Wang, Ying-Chun Yu, Hsin-Yuan Chen, Yi-Fen Chiang, Mohamed Ali, Tzong-Ming Shieh, Shih-Min Hsia

**Affiliations:** 1 Department of Nursing, Ching Kuo Institute of Management and Health, Keelung 20301, Taiwan; d49505002@gm.ym.edu.tw; 2 School of Nutrition and Health Sciences, College of Nutrition, Taipei Medical University, Taipei 11031, Taiwan; hsin246@gmail.com (H.-Y.C.); yvonne840828@gmail.com (Y.-F.C.); 3 Sex Hormonal Research Center, Department of Obstetrics and Gynecology, China Medical University Hospital, Taichung 40403, Taiwan; yingchun.ycyu@gmail.com; 4 Graduate Institute of Biomedical Sciences, Center for Tumor Biology, School of Medicine, China Medical University, Taichung 40403, Taiwan; 5 Clinical Pharmacy Department, Faculty of Pharmacy, Ain Shams University, Cairo 11566, Egypt; mohamed.aboouf@pharma.asu.edu.eg; 6 School of Dentistry, China Medical University, Taichung 40403, Taiwan; tmshieh@mail.cmu.edu.tw; 7 Graduate Institute of Metabolism and Obesity Sciences, College of Nutrition, Taipei Medical University, Taipei 11031, Taiwan; 8 School of Food and Safety, Taipei Medical University, Taipei 11031, Taiwan; 9 Nutrition Research Center, Taipei Medical University Hospital, Taipei 11031, Taiwan

**Keywords:** licorice, cancers, adverse effects, chemotherapy, radiotherapy

## Abstract

Cancers represent a significant cause of morbidity and mortality worldwide. They also impose a large economic burden on patients, their families, and health insurance systems. Notably, cancers and the adverse reactions to their therapeutic options, chemotherapy and radiotherapy, dramatically affect the quality of life of afflicted patients. Therefore, developing approaches to manage chemotherapy- and radiotherapy-induced adverse reactions gained greater attention in recent years. *Glycyrrhiza glabra* (licorice), a perennial plant that is one of the most frequently used herbs in traditional Chinese medicine, has been heavily investigated in relation to cancer therapy. Licorice/licorice-related regimes, used in combination with chemotherapy, may improve the adverse effects of chemotherapy. However, there is little awareness of licorice-containing herbs alleviating reactions to radiotherapy and chemotherapy, or to other induced adverse reactions in cancer treatment. We aimed to provide a descriptive review, and to emphasize the possibility that licorice-related medicines could be used as an adjuvant regimen with chemotherapy to improve quality of life (QoL) and to reduce side effects, thus, improving compliance with chemotherapy. The experimental method involved searching different databases, including PubMed, the Cochrane Library, and Wang Fang database, as of May 2022, to identify any relevant studies. Despite a lack of high-quality and large-scale randomized controlled trials, we still discovered the potential benefits of licorice-containing herbs from published clinical studies. These studies find that licorice-containing herbs, and their active ingredients, reduce the adverse reactions caused by chemotherapy and radiotherapy, and improve the QoL of patients. This comprehensive review will serve as a cornerstone to encourage more scientists to evaluate and develop effective Traditional Chinese medicine prescriptions to improve the side effects of chemotherapy and radiation therapy.

## 1. Introduction

**Figure 1:**
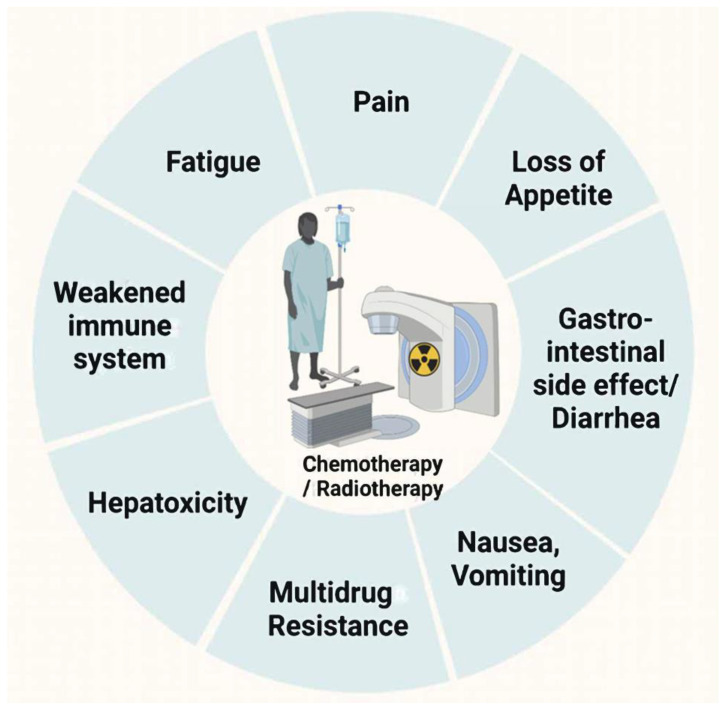
Side effects of chemotherapy and radiotherapy.

Cancer is one of the leading causes of premature death and a significant barrier to increasing life expectancy in almost every country in the world. In 2020, 19.,3 million new cancer cases were estimated, as well as almost 10 million cancer deaths [1]. Traditionally, standard cancer therapies include surgery, cytotoxic chemotherapy, and radiation therapy. Nevertheless, oncology treatment regimens, especially for chemotherapy/radiotherapy, may lead to other complications, such as fatigue (50~90%), chronic pain (50~70%), oral mucositis, anorexia (~85%), gastrointestinal toxicity, hepatotoxicity, nephrotoxicity, insomnia (30~60%), edema, depression/anxiety (24%/24%), or constipation (30~80%) [2,3,4,5]. These complications may decrease therapeutic compliance, lead to treatment interruption, or reduce the quality of life (QoL) (Figure 1) [6]. Nowadays, pharmacologic antiemetic therapy has become a major supportive care initiative [7]; however, its therapeutic effects remain extremely limited in improving vomiting and other complications. It is, therefore, important to develop more effective therapeutic strategies, or adjuvant treatments, to synergistically enhance the efficacy and reduce the toxicity of chemotherapy and radiotherapy. 

After undergoing chemotherapy/radiation, quality of life (QoL) is impacted by several symptoms: vomiting/nausea, diarrhea/constipation, and immunodeficiency. 

The chemical compounds of chemotherapy, largely purified from plants, are important for cancer treatment [8]. Cancer relapses, one of the major causes of death in cancer, occur because cancers develop resistance to classical chemotherapeutic agents, or even novel targeted drugs, over time. Therefore, it is essential to discover more therapeutic agents [9]. Moreover, some natural products also possess the ability to reduce the complications induced by chemotherapy and radiotherapy.

Recent studies demonstrate that licorice, as well as licorice-purified compounds, has the potential to abrogate the onset and progression of different malignancy cancers, both in vitro and in vivo [10,11,12,13,14,15]. Moreover, previous studies also suggest that licorice is a beneficial medicine plant used as a cure for nausea and vomiting [16]. In contrast, consuming excessive quantities of licorice is also associated with hypertension, hypokalemia, cardiac arrhythmias, and metabolic alkalosis. These effects may be due to the alternation of the renin–angiotensin–aldosterone system [17,18]. However, the present review aims to make an overview of the licorice-containing herbs that have the potential to alleviate the adverse reactions induced by radiotherapy and chemotherapy.

## 2. Utility of Licorice-Containing Herbs in Cancer

### 2.1. Licorice Introduction

Licorice belongs to the genus *Glycyrrhiza*, and *radix glycyrrhizae* (RG) is the dried roots and rhizomes of licorice. Licorice is commonly used as a natural sweetener and in herbal medicine. It mainly acts as a supplement in Western countries, for products such as herbal teas, soft drinks, and tobacco products. However, it is regarded as a medicine in Asia. Licorice is utilized to relieve pain, phlegm, spasms, cough, and dyspnea. The abundant active ingredients in licorice demonstrate efficacy in many different biological and physiological functions. To date, more than 300 bioactive compounds have been identified in licorice, including ~100 types of triterpenoid saponins and sapogenins, and ~300 kinds of phenolic compounds [17]. However, it has been found that the cultivated geographical area, the state of plant maturity, environmental conditions (including the pH of the soil, temperature, and weather), harvesting, and processing all affect the content of the bioactive compounds in licorice [18]. For example, the triterpenoid saponins in licorice, especially glycyrrhizic acid (GL; approximately 1.84% to 9.82% of licorice, depending on the sources and methods of extraction), are the major constituents and bioactive ingredients of licorice [19,20,21]. Flavonoids (approximately 1.78% to 4.82% of licorice, depending on the sources and methods of extraction) are the other main bioactive compound found in licorice, including isoliquiritigenin (ISL), isoliquiritin, and liquiritigenin, etc. [19,22]. In the 2010 edition of the Chinese Pharmacopoeia, GL and isoliquiritigenin were selected as the biomarkers for licorice, and it is stated that their content should exceed 2% and 0.5%, respectively [23]. Interestingly, we found that these two major compounds (GL and ISL) appear in many studies related to chemotherapy, which will be discussed further in a later section.

Licorice can be simply categorized into three Glycyrrhiza species: *Glycyrrhiza uralensis Fisch*., *Glycyrrhiza glabra L*., and *Glycyrrhiza inflata Bat*. [24]. In China, *G. uralensis*, *G. glabra*, and *G. inflata* are considered equivalent, and are combined and utilized as licorice without discrimination in the 2015 edition of the Chinese Pharmacopoeia. However, the morphological characteristics of the three Glycyrrhiza species show differences in the root, rhizome, seed, fruit, and inflorescence, as well as in the leaf and stem height. It is difficult to identify these licorice species accurately based only on their root or rhizome morphology [25].

There are significant differences between the species, which were established by the analytical methods of numerous studies aimed at separating and quantifying the active ingredients in licorice samples. These studies report that different licorice species have species-specific markers (Table 1); for example, the content of major flavonoids (liquiritin, liquiritigenin, and isoliquiritin) in *G. uralensis* is higher than that in *G. glabra*, and glycycoumarin only exists in *G. uralensis* [26,27,28,29,30]. Glycyrrhizin, 50 times sweeter than sugar and especially suitable for children, is evenly distributed in the three species [29]. The amount of isoliquiritigenin (2′,4′,4-trihydroxychalcone, ISL), one of the major bioactive compounds in licorice, is higher in *G. uralensis* than in *G. glabra* and *G. inflata*. [31,32]. 

### 2.2. Chemopreventive Activities of Licorice

Licorice and its derivatives exert anti-inflammatory and antioxidant effects, suggesting their potential as chemopreventive or therapeutic agents. For example, licorice can be used for reducing inflammation and allergic responses, as well as preventing liver damage [24,33,34]. Licorice extract also acts as a moderate hypocholesterolemic nutrient, and a potent antioxidant agent, to prevent cardiovascular disease [35]. Clinical data suggest that licorice, or its bioactive components, prevent dyspepsia and hyperlipidemia [36,37]. When taken concurrently with a glycyrrhizin-containing product, licorice is shown to afford hepatoprotection during alcohol consumption [38]. Three randomized clinical trials claim that Glycyrrhiza acts as a mucoadhesive film, improving oral mucositis during radiotherapy [39,40,41]. Moreover, preoperative gargling with a licorice solution reduces postoperative sore throat, thus, revealing its analgesic properties [42]. The clinical trials of licorice are summarized in Table 2.

### 2.3. Licorice Literature Search Strategy

#### 2.3.1. Review Purpose

Licorice/licorice-related medicine, combined with chemotherapy, may potentially reduce the side effects of chemotherapeutics, such as mucositis, anemia, anorexia, and fatigue, and may offer cheaper and safer options than current conventional medication. In this review, we mainly focused on the use of licorice-related medicines, including licorice itself, licorice-mixed ingredients (prescriptions/Kampo), and purified compounds, in an attempt to summarize the current knowledge regarding licorice application in chemotherapy.

Our review mainly targets the questions below:

- Does licorice/licorice-related medicine/purified compound combined with chemotherapy improve the adverse effects of chemotherapy?
- What type of adverse effects are suitable for treatment with licorice-related medicine?
- Do they have recorded adverse effects?
- Does the additional application the improve quality of life (QoL) among those receiving conventional chemotherapy?

#### 2.3.2. Search Database

The following databases were searched: PubMed, the Cochrane database, and Wang Fang database (in Chinese). In PubMed and the Cochrane database, references were included from the inception of each database to the end of May 2022. In vivo studies (clinical trials) were collected from the three databases. In vitro studies were mainly obtained from PubMed. To obtain the relevant studies, a three-step search strategy was followed. First, the “Title/Abstract” mode in the search engine was used. Next, a search of the identified keywords and index terms was undertaken across all included databases. For licorice, for example, “licorice[Title/Abstract]” was searched, then the second keyword was added “(licorice[Title/Abstract]) AND chemotherapy[Title/Abstract])”. Finally, clinical trials were found based on the previous search.

For licorice-related clinical trials, the following medical subject heading (MeSH) terms and keywords were used for the search: (licorice OR Glycyrrhiza) AND (cancer OR chemotherapy) AND (chemotherapy side effect OR mucositis OR anemia OR anorexia OR fatigue) AND (clinical trial), with slight modifications for individual searches to suit the instructions of different databases. The above terms were searched in Chinese characters and pinyin (Wang Fang database).

The searching strategy was more complicated for licorice-related medicine (also called Kampo or prescription). The same prescription can have different names in different countries. Taking TJ-41 as an example, hochuekkito, hochu-ekki-to, and TJ-41 are the common names in Japan, while it is known as bojunikgi-tang (bojungikki-tang) in Korea, and bu-zhong-yi-qi tang in China. Interestingly, with/without a hyphen in the name during the search leads to other results in the literature. The different names of each licorice-related medicine were checked in the databases, and the information is summarized in Table 3 and Table 4, respectively. Besides the name of the medicine, the following medical subject heading (MeSH) terms and keywords were applied for the search: AND (cancer OR chemotherapy) AND (mucositis OR anemia OR anorexia OR fatigue) AND (clinical trial). As above, the terms were searched in Chinese characters and pinyin (Wang Fang database). 

The inclusion criteria were based on the following:

(1) Type of participant: cancer patients treated with chemotherapy or radiation therapy;
(2) Type of study: We tried to include as many as possible. As the number of licorice-related clinical trials is low, the study size is small-scale;
(3) Type of intervention: Participants in the intervention groups were treated with licorice or licorice-related medicine combined with chemotherapeutic drugs. There was no concern about the forms of interventions (e.g., decoction, capsule, acupoint patch gel, and granule), the dosage, or the treatment duration. The control groups used chemotherapy alone, chemotherapeutic drugs plus a placebo, or chemotherapeutic drugs plus western medicine. The control groups used chemotherapy alone, chemotherapeutic drugs plus a placebo, or chemotherapeutic drugs plus western medicine;
(4) Type of outcome measure: Mainly focusing on chemotherapy-induced side effects, such as fatigue, oral mucositis, anorexia, anemia, constipation, etc.

The exclusion criteria were as follows:

(1) The study purpose is not related to chemotherapy-induced side effects;
(2) Duplicate studies in a different database, review, animal experiments, and conference abstracts;
(3) Misunderstandings, misleading studies, and inappropriate use/measurement;
(4) Studies did not present clearly, including an inappropriate or unclear study design to collect data;
(5) Lacking statistical analysis.

##### Description of Review Context

A total of 48 trials were included in this review; all of these trials took place in Asia (in China, Japan, Taiwan, and Korea) and were reported in English or Chinese. The details of the mechanism and the clinical trials are summarized in Table 3 and Table 4, respectively. A descriptive review is presented.

**Table 1:** Most common *Glycyrrhiza* species used as medicine.

Glycyrrhiza Species	Region	Specific Content	Ref
*Glycyrrhiza uralensis (Glycyrrhiza radix)*	China Northeastern Far east Russia	Owning the highest content of flavonoids (liquiritin, liquiritigenin, and isoliquiritin). Glycycoumarin only represented in *G. uralensis*.	[26,27,28,29,30,43]
		Isotrifloliol, licoricone, neoglycyrol, glycyrin, and licorisoflavan A in *G. uralensis* are higher.	[26]
		Glyinflanin D/G and licoflavone B are absent.	[44]
*Glycyrrhiza glabra*	Italy Spain China Russia Iran Central Asia	Owning the highest content of 18α-glycyrrhizic acid and 18β-glycyrrhizic acid.	[45]
		Higher content of saponins–licorice saponin K2/H2, licorice saponin B2, and licorice saponin G2/yunganoside K2. Quercetin absent in *G. glabra*.	[44,46,47]
		The highest content of apiosides (liquiritin apioside, isoliquiritin apioside, licuraside).	[30]
		Abundant 8-cyclized isoprenyl isoflavanes (e.g., glabridin and 4′-O-methylglabridin).	[29]
		Polysaccharide content in *G. glabra* is the highest.	[48,49]
*Glycyrrhiza*	China, Asia	Highest content of triterpene saponins.	[25,29]
*inflata*		Chalcone derivatives such as licochalcone (A, B, C, E), kanzonol C, and echinatin in *G. inflata* are higher.	[29,44,50]
		The content of quercetin is higher than that in *G. uralensis*.	[44,46,47]
		Highest content of prenylated chalcones.	[44]

**Table 2:** Licorice and its components applied in chemopreventive clinical trials.

Name	Disease/Disorder	Dose/Duration	Trial				Location/ Identifier No.	Ref
			Patient (*n*)	Experiment Group	Control Group	Outcome		
Extract of *G. glabra*	Radiotherapy Head or neck	Oral 100 c.c/Bid 2 weeks	*n* = 37	Extract of *G. glabra*	Placebo (radiotherapy)	Prevent oral mucositis	IRCT201203012464N4 Iran Tehran University of medical science	[39]
*G. glabra* (yashtimadhu)	Radiotherapy Head or neck	Oral 5 g/Bid 6 weeks	*n* = 127	*G. glabra*	Placebo (radiotherapy)	Prevent oral mucositis	Himalayan Institute of Medical Sciences, Dehradun, India	[40]
Licorice	Radiotherapy Head or neck	Mouth wash	*n* = 60	Licorice mucoadhesive film	Placebo mucoadhesive film	Prevent oral mucositis	Isfahan University of Medical Sciences, Isfahan, Iran	[41]
Licorice extract	Randomized Double-blind	Oral 1 g/Tid	*n* = 236	+licorice extract	Sugar water	Pain relieving	NCT02968823	[42]
Licorice	Dyspepsia	380 mg/Bid 4 weeks	*n* = 120	+licorice	N.A.	Improved *H. pylori* eradication	IRCT2014061718124N	[36,37]
Glycyrrhizin	Alcohol consuming	Oral 0.1–0.3% 12 days	*n* = 24	+Licorice	Placebo (alcohol)	Hepato-protection	N.A.	[38]

When Kampo medicine is combined with chemotherapy/radiation, it reduces the side effects induced by chemotherapy. For example, TJ-48 improves cancer-related fatigue, anorexia, and anemia. TJ-43 also mitigates anorexia and chemotherapy-induced GI problems. TJ-41 mainly acts on fatigue and mucositis.

## 3. Traditional Herbal Formulation

**Figure 2:**
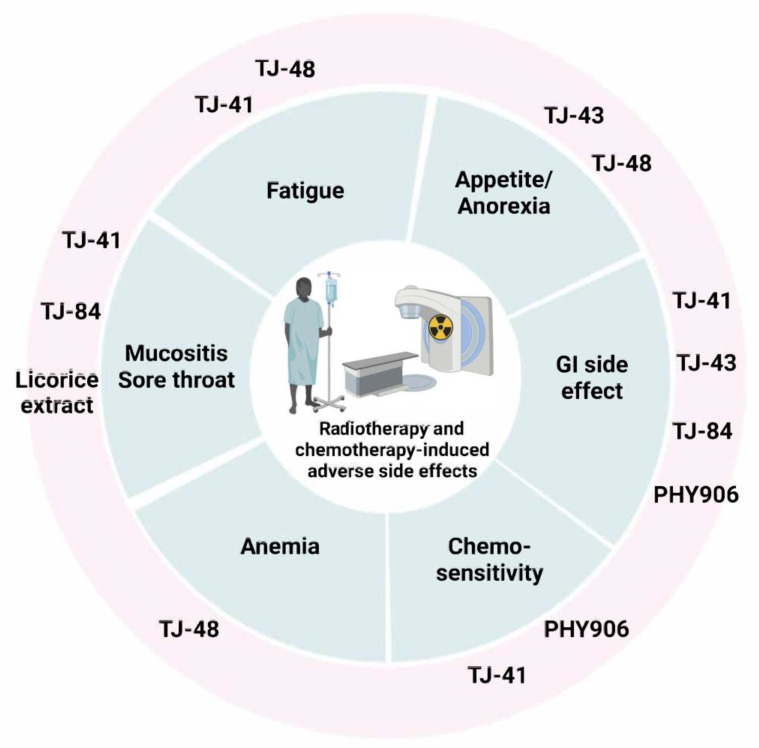
Kampo combinations improve chemotherapy-induced side effects in clinical trials.

Traditional Chinese medicine (TCM), called Kampo in Japan, comprises a combination of compatible natural herbs. The Japanese Ministry of Health, Labor and Welfare officially approved the wide use of traditional herbal formulations as ethical pharmaceutical products [51,52]. Chinese herbal products are commonly applied for daily practice as dietary supplements. In China, numerous clinical studies explore TCM as an adjuvant cancer treatment during chemotherapy or radiotherapy [53,54]. TCM alleviates the toxicities of chemotherapy agents and controls the side effects, thus, improving compliance with chemotherapy and the patient’s QoL [6]. Licorice-related TCM contains many phytochemicals, including triterpenoid saponins, flavanones, chalcones, and coumarins and their glycosides, which exhibit a broad range of biological activities [55,56,57]. Here, we summarize a brief outline of the pharmacological effects of TCM containing licorice in Table 3, and the related clinical trials in Table 4 (Figure 2).

### 3.1. TJ-84

Daiokanzoto (DKT; also called da-huang-gan-cao-tang or TJ-84) is a Kampo medicine that is composed of crude extracts of rhubarb rhizomes and Glycyrrhiza roots. TJ-84 is mainly investigated for use on peptic ulcers, hepatitis C, and pulmonary and skin diseases [58,59,60,61]. Wang et al. study the chemical constituents in TJ-84, using the combination of LC–ESI-Q-TOF–MS and LC–ESI-IT–MS, and identify 104 compounds [62]. Flavonoids and triterpenoids are the major bioactive compounds present in Glycyrrhizae radix, and they exhibit diverse biological effects [63]. Furthermore, Takayama et al. identify that in Sennoside A (SA), rhein 8-O-β-D-glucopyranoside from rhubarb, and liquiritin (LQ) from glycyrrhiza, are the main active constituents in vivo that alleviate constipation [58,59,64,65]. TJ-84 exerts a purgative activity that may mediate gut microbiota against constipation [59,65], and clinical data reveal its effectiveness against constipation, vomiting, and nausea [59,64,65,66,67,68]. A 2019 retrospective cohort study confirms the same effect on constipation [69]. It is reported that TJ-41 possesses anti-inflammatory potential by mediating the secretion of pro-inflammatory cytokines (IL-6 and CXCL8) and tissue degrading enzymes matrix metalloproteinase (MMP-1 and MMP-9), in addition to preventing biofilm formation [70]. In another in vitro study, Yoshida et al. demonstrate that TJ-41 attenuates 5-fluorouracil-induced apoptosis, by inhibiting the production of mitochondrial reactive oxygen species [66]. This leads to the suggestion that TJ-41 could be used to treat oral mucositis in patients receiving chemotherapy [71]. A clinical study confirms that TJ-84 improves mucositis in esophageal cancer patients treated with chemotherapy [61]. 

### 3.2. TJ-41

Bu-zhong-yi-qi-tang (hochuekki-to or TJ-41 in Japanese, or bojungikki-tang in Korean) is prescribed to treat fatigue, visceroptosis, and chronic diarrhea. It contains seven herbs, including Pinellia tuber, Scutellaria baicalensis, Zingiberis rhizoma, Zizyphi fructus, Coptidis rhizoma, Glycyrrhiza radix, and Panax ginseng. Using High performance liquid chromatography-diode array detection (HPLC-DAD) guidance, liquiritigenin and ISL were found in the highest abundance in TJ-41 [72]. When TJ-41 is used to treat cancer patients after chemotherapy, with medication such as cisplatin or 5-FU, it improves chemosensitivity and inflammation [73,74]. Regarding cancer-related fatigue, Kuroda et al. (1985) demonstrate that treatment with TJ-41 results in a significant improvement in the quality of life (QoL) of 162 patients with cancer-related cachexia (i.e., anorexia and fatigue), especially targeting fatigue and anorexia [75]. In another pilot randomized clinical trial, the use of TJ-41 results in an improvement in chemotherapy-induced fatigue, without any significant adverse effects [76]. Based on the above, TJ-41 is a common prescription medicine for cancer patients in Japan, and its use results in improvements in cancer-related fatigue and quality of life in these patients [74,75]. As far as the molecular mechanism is known, TJ-41 may be attributed to peripheral immunity, suppressing the immune escape of tumors, and inducing immune deterioration [75,76,77]; for example, inhibiting the production of proinflammatory cytokines, particularly IL-6 [78]. Furthermore, TJ-41 may inhibit Tumor Necrosis Factor-α (TNF-α), Interleukin-6 (IL-6), IL-10, Transforming Growth Factor-1 (TGF-1), and Interferon (INF) production, in order to combat chronic fatigue syndrome [78,79,80]. During the search of the literature, we also found some clinical studies in Japan and China [81,82,83,84,85,86,87,88,89] that apply TJ-41 with chemotherapy, with the studies suggesting that TJ-41 reduces the adverse effects of chemotherapy and improves the overall QoL scores. A multicenter randomized phase II study from 2019 [90], targeting stage II/III gastric cancer patients with chemotherapy (*n* = 113), also supports the same suggestion [90]. Another study, in the form a retrospective review, includes 1412 advanced non-small cell lung cancer (NSCLC) patients divided into two groups. The control group received cisplatin treatment; while the other was treated with cisplatin and TJ-41. The results suggest that TJ-41 improves chemosensitivity, immunity, adverse effects, and overall QoL [91]. In summary, TJ-41 has potent immunomodulatory, anticancer, and fatigue-reducing actions.

### 3.3. TJ-43

Liu-jun-zi-tang (TJ-43 or rikkunshito in Japanese, yukgunja-tang in Korean) includes six herbs: Ginseng Radix, Poria cocos, Rhizoma atractylodis macrocephalae, licorice root, pinelliae tuber, pericarpium citri, common ginger, and jujube. TJ-43 is often prescribed for anorexia and upper gastrointestinal (GI) disorders [92,93]. Based on a components assay, TJ-43 shows 36 active components, with glycycoumarin, nobiletin, tangeretin, ISL, [8]-shogaol, glycyrrhetic acid, and hesperetin being dominant [94]. Some functioning active components are reported in TJ-43, such as hesperidin, ISL [95,96], and glycycoumarin [97]. In a preclinical study, high levels of liquiritigenin and its glycoside forms are identified in plasma and in the brain after a single oral administration of TJ-43 (1000 mg/kg) [98]. ISL is not only detected after the oral administration of TJ-43, but a comparable amount of ISL is also shown in plasma and brain distribution, especially compared with an ISL (5 mg~50 mg/kg) single treatment [94]. Magomi et al. suggest that ISL is one of the most important active components in TJ-43, acting as an antagonist to both CRF receptor 1 (CRFR1) and serotonin 2C receptor (5-HT2CR) in order to reduce ghrelin secretion, which further improves stress-induced anorexia [94,95,99]. In addition, several multi-center, double-blinded, randomized placebo-controlled studies were conducted to examine its effect with regard to improvements in upper GI disorders, such as dyspepsia [100,101,102,103]. Another randomized, controlled trial, pilot study (*n* = 40) was conducted by Ko et al. to estimate the efficacy and the safety of TJ-43 [104,105]. Generally speaking, chemotherapy-induced nausea and vomiting (CINV) occurs within 24 h after chemotherapy administration. Nausea and vomiting are two of the most common side effects experienced by patients [106]. Although the mechanism of chemotherapy-induced CINV is not thoroughly understood, cisplatin-related adverse effects are associated with the secretion of serotonin (5-hydroxytryptamine, 5-HT) and changes in ghrelin dynamics [107]. Therefore, 5-HT3 receptor antagonists are widely applied to prevent cisplatin-induced CINV. Even with a standard treatment, a three-drug combination of a 5-HT3 receptor antagonist, a neurokinin-1 receptor antagonist, and dexamethasone (as an aprepitant regimen) show only a 60~80% mitigation of CINV [107]. Seeking further improvement in CINV, some clinical studies recommend the co-administration of TJ-43 as an effective option for breakthrough CINV, as TJ-43 also acts as an antagonist at the 5-HT2B/C, 5-HT3 receptors and the ghrelin receptor [95,99,108,109,110]. TJ-43 combined with chemotherapy is shown to prevent CINV in esophagus, gastric, lung, cervical, and corpus cancer patients [111,112,113,114]. In addition, anorexia (loss of appetite) is also a common concomitant of cancer, especially in advanced cancer. Anorexia may be induced by cancer itself and cancer treatment. Both chemotherapy and radiation therapy can trigger varying degrees of anorexia. A few randomized controlled trials aimed to evaluate the efficacy and safety of TJ-43 for chemotherapy-induced anorexia or cancer-induced anorexia [104,105,114]. Many clinical studies indicate that TJ-43, co-administered with chemotherapy, improves anorexia and aids in maintaining food intake by mediating the 5-HT2B/C receptor or the ghrelin receptor [104,105,109,111,112,113,114,115,116,117]. Based on the above description, it is suggested that TJ-43 application mainly acts on CINV and anorexia. This conclusion was reached by different research groups (in China, Japan, and Korea) using the same receptors. 

TJ-43, also called li-jun-zi-tang (LJZT) in China, has a long history of use for functional dyspepsia. The prescription originated hundreds of years ago (in the Ming Dynasty) in China. However, even though there are many clinical trials studying chemotherapy combined with TJ-43, only a small number of trials use validated international criteria to assess the side effects of chemotherapy. Many of them present with a considerable risk of bias. Based on these concerns, we only included three studies in this review [118,119,120,121,122]. 

Overall, all the relevant studies suggest that TJ-43 combined with chemotherapy can mitigate chemotherapy-induced CINV and anorexia.

### 3.4. TJ-48

Shi-quan-da-bu-tang (juzentaiho-to or TJ-48 in Japanese, or sipjeondaebo-tang in Korean) can be used to treat various kinds of diseases, such as anemia, rheumatoid arthritis, atopic dermatitis, chronic fatigue syndrome, and ulcerative colitis. TJ-48 is composed of 10 herb components, including Ginseng radix, Astragaliradix, Angelicae radix, Rehmanniae radix, Atractylodis lanceae rhizoma, Cinnamomi cortex, Poriacocos, Paeoniaeradix, Ligustici rhizoma and Glycyrrhizae radix. There are 36 major components in TJ-48, including flavonoids/flavonoid glycosides (liquiritin, isoliquiritin, etc.), triterpenoid saponins, iridoid glycosides, phthalides, and phenylpropionic acids, which were characterized using high-performance liquid chromatography / electrospray ionization mass spectrometry (HPLC–ESI–MS/MS) [123]. A study by Tsuchiya et al. (2008) suggests that TJ-48 functions as a hepatic protectant by hampering Kupffer cell-induced oxidative stress [124]. When Kupffer cells are inhibited, TJ-48 plays an important role in lowering pro-inflammatory cytokines and oxidative stress levels. A preclinical study shows that TJ-48 inhibits the production of IL-6, MCP-1, PYY, and GLP-1 and ameliorates cancer-induced anemia using a CT-26 tumor-bearing mouse cancer anorexia/cachexia model [125]. Overall, TJ-48 may be a better option for treating cancer associated with anorexia. TJ-48 is one of the most commonly used traditional herbal medicines in Asia, and is prescribed for patients who suffer from anemia, fatigue, and anorexia. Anorexia may result from the cancer, chemotherapy, or radiotherapy. A study implies that TJ-48 used as a treatment for anorexia regulates the levels of glucagon-like peptide-1 (GLP-1) and peptide tyrosine tyrosine (PYY), which are satiety stimulators [125]. Recently, two clinical studies apply TJ-48 as a treatment for anorexia to evaluate its efficacy [126,127]. Besides anorexia, TJ-48 ameliorates anemia [128], assisting patients receiving chemotherapy [129]. Fatigue is another frequent complaint after chemotherapy; thus, a few clinical studies related to fatigue were conducted [81,125,130]. In a study by Tsuchiya et al., which includes 48 hepatocellular carcinoma (HCC) patients treated with TJ-48 and then followed up for up to six years, it is suggested that TJ-48 improves HCC recurrence-free survival [124]. Concerning long-term survival, this tentative conclusion needs further detailed investigation in the future. 

### 3.5. PHY906

PHY906 (YIV-906 or KD018) is derived from huangqin tang (HQT), which is composed of four herbs: Glycyrrhiza uralensis Fisch (G), Paeonia lactiflora Pall (P), Scutellaria baicalensis Georgi (S), and Ziziphus jujuba Mill (Z). Huangqin tang was first documented in Chinese texts 1800 years ago as being used in the treatment of GI disorders, including diarrhea, nausea, and vomiting, and abdominal cramps. Based on LC–MS analysis, its chemical profile contains 64 bioactive compounds, including flavonoids, triterpene saponins, and monoterpene glycosides [131]. One of the main components with anticancer activity is ISL. The preparation of PHY906, packaged in capsules, is conducted according to the current Good Manufacturing Practices (cGMPs), under the United States Food and Drug Administration (FDA) guidance. PHY906 demonstrates chemotherapeutic efficacy enhancement with a variety of anticancer agents in various cancers. PHY906 clinical trials indicate that PHY906 acts as an adjuvant to CTP-11, 5-fluorouracil (5-FU), leucovorin, and capecitabine in the treatment of advanced colorectal, pancreatic, and liver cancer [132,133,134,135,136,137,138,139,140,141]. PHY906 has additive antitumor activity, but does not alter the pharmacokinetics or toxicity of irinotecan, 5-FU, and leucovorin [134]. In a phase II clinical trial study, it is found that PHY906 enhances the capecitabine-induced antitumor activity and survival rate in Asian patients with hepatocellular carcinoma (HCC) and hepatitis B virus infection (*n* = 39) [142]. PHY906 plus capecitabine provides a safe and feasible salvage therapy in pancreatic cancer patients (*n* = 25) [135]. A combination of PHY906 and capecitabine could be an effective therapy elevating the median survival of Asian patients suffering from hepatocellular cancer [138]. PHY906 (800 mg BID) increases the therapeutic index of capecitabine in patients with advanced stage disease (APC) and other GI malignancies, by reducing side effects such as diarrhea (*n* = 24) [137]. Clinical trial results consistently suggest that PHY906 is one of the most extensive chemotherapeutic adjuvants. Advanced clinical trials (phase III) are needed to demonstrate the effectiveness of PHY906 as an adjuvant therapy for cancer patients undergoing chemotherapy.

**Table 3:** Common Kampo prescriptions (including *Glycyrrhiza*) for cancer therapy.

Name of Kampo	Other Name	Composition	Biological Activity/Treatment	Evidence of the Activity	Ref.
TJ-84	Daiokanzoto (in Japanese) Da-huang-gan-cao-Tang (in Chinese)	Includes 2 herbs: *Rhubarb and Glycyrrhiza*	Constipation Purgative activity Mucositis	Preclinical: (i) Purgative activity inhibits periodontopathogen via NF-κB pathway; (ii) reduces the secretion of pro-inflammatory cytokine (IL-6 and CXCL8) production; (iii) inhibits MMP-1 and MMP-9 catalytic activities, contributing to anti-inflammation; (vi) decreases AQP3 expression attributed to gut microbiota homeostasis; (v) attenuates 5-FU-induced cell death through the inhibition of mitochondrial ROS production. Clinical: (i) Alleviates cancer-related fatigue. Reduces adverse reactions to radiotherapy or chemotherapy; (ii) improves constipation (double-blind test in Japan); (iii) improves mucositis in esophageal cancer when combined with chemotherapy.	[58,59,60,61,64,66,69,70]
TJ-41	Bu-zhong-yi-qi tang (BZYQ) (in Chinese) Hochu-ekki-to (in Japanese) Bojungikki- tang (in Korean)	Includes 7 herbs: *Pinellia tuber, Scutellaria baicalensis, Zingiberis* *hizome, Zizyphi fructus, Coptidis* *hizome, Glycyrrhiza radix, and Panax ginseng*	Immunomodulation Anti-tumor Anti-fatigue	Preclinical: (i) Reverses cisplatin resistance through induction of apoptosis and autophagy in lung cancer cells; (ii) inhibits 5-FU-induced intestinal mucositis via the suppression of inflammatory cytokine upregulation; (iii) increases lymphocyte cell-surface antigens: CD3^+^-cells and CD3^+^/CD4^+^ cells; (iv) inhibits TNF-α, IL-6, IL-10, TGF-1 and INFγ against chronic fatigue. Clinical: (i) Protective effect of intestine and hematopoietic organs against radiation damage; (ii) improves localized radiotherapy-induced immune deterioration; (iii) improves cancer-related fatigue and QOL; (iv) reduces radiation- or chemotherapy-induced adverse effects.	[73,74,75,76,79,80,143,144,145]
TJ-43	Rikkunshi-to (in Japanese) Liu-jun-zi tang (in Chinese) Yukgunja-tang (in Chinese)	Includes 6 herbs: *Ginseng radix, Poria cocos, Rhizoma atractylodis macrocephalae, Glycyrrhizae radix et rhizoma, Pinelliae rhizoma, Pericarpium citri, common ginger, and Jujube.*	Dyspepsia Anorexia Chemotherapy-induced nausea and vomiting (CINV) Appetite	Preclinical: (i) Improves cisplatin-induced anorexia (decreases plasma-acylated ghrelin level and enhances food intake) by acting as antagonists at the 5-HT2B/2C receptors. Clinical: (i) Improves CINV by mediating 5-HT2B/2C receptors and ghrelin receptor signaling; (ii) gastroprotective actions: enhances gastric motility through the 5-HT3 receptor-antagonistic effect; (iii) appetite-stimulating effect via mediating ghrelin receptor signaling (blocked by (D-Lys3)-GHRP-6).	[95,100,101,102,103,104,108,109,111,112,117,146,147,148,149,150]
TJ-48	Shi-quan-da-bu-tang (in Chinese) Juzen-taiho-to (in Japanese)	Includes 10 herbs: *Ginseng radix, Astragali radix, Angelicae radix, Rehmanniae radix, Atractylodis lanceae rhizoma, Cinnamomi cortex, Poria, Paeoniae radix, Ligustici rhizoma Glycyrrhizae radix*	Anti-tumor Immunomodulation Periodontal disease	*Preclinical*: (i) Alleviates bone marrow suppression caused by TS-1 in mice; (ii) reduces pro-inflammatory cytokines and oxidative stress in the liver; (iii) inhibits the production of IL-6, MCP-1, PYY and GLP-1; (iv) anti-tumor via enhanced CD8^+^ T cell-mediated immunity in CD1d^−/−^ mice lacking NKT cells. *Clinical*: (i) Regulates T cells: decreases Foxp3^+^ Treg populations; (ii) inhibits B16 cell metastasis by inducing NK cell activity; (iii) inhibits osteoclast differentiation.	[124,125,143,144,151]
PHY906	KD018, YIV-906 Huang-qin-tang (HQT)	Includes 4 herbs: *Scutellaria baicalensis Georgi, Paeonia lactiflora Pall, Glycyrrhiza uralensis Fisch, Ziziphus jujuba Mill*	Anti-tumor Anti-inflammatory	*Preclinical:* Enhances the antitumor activity of Sorafenib in nude mice bearing HepG2 xenografts, by targeting the inflammatory state of the tumor tissue microenvironment. Alleviates chemotherapy-induced side effects, such as diarrhea. *Clinical:* Enhances the antitumor efficacy of some anticancer drugs, but also alleviates chemotherapy or targeted therapy (e.g., CTP-11)–induced side effects.	[132,133,134,135,136,141]

**Table 4:** Cancer-related clinical trial of Chinese herbal medicines that include *Glycyrrhiza*.

Name of Kampo	Disease/Disorder	Dose/Duration	Trial				Location/Identifier No.	Ref
			Patient (n)	Experiment Group	Control Group	Outcome		
TJ-84	Esophageal cancer	Oral Tid 2.5 g/bag	*n* = 15	*n* = 7 +TJ-84	*n* = 9 DFP * therapy	A beneficial effect for oral health.	Tokushima University Hospital, Japan	[61]
	Nasopharyngeal carcinoma	Acupoint patch on the skin	*n* = 60	*n* = 30 +TJ-84	*n* = 30 ** (Cisplatin)	Improves CINV and constipation.	Jiangxi Provincial People’s Hospital, China	[67]
	NSCLC	Acupoint patch on the skin	*n* = 116	*n* = 60 + TJ-84	*n* = 56 **		Zhongshan Hospital, Shanghai, China	[68]
TJ-41	Cancer-related fatigue	Oral 2.5 g/Tid 2 weeks	*n* = 40	*n* = 20 TJ-41	*n* = 20	Improves fatigue (experimental group vs. control group, *p* < 0.05)	Kyung Hee University (Korea) KHU-20090596 (Completed)	[76]
	Cancer-related-fatigue	Oral 3.7 g/Bid 2 weeks	*n* = 112	*n* = 56	*n* = 56	No result yet.	Started Oct 2020 KCT0004967 (Ongoing)	[152]
	Advanced NSCLC	Oral	*n* = 92	*n* = 46 TJ-41	*n* = 46 Chemotherapy	Improves chemosensitivity, QoL and adverse effects of chemotherapy.	Changsha Traditional Chinese Medicine Hospital, China	[82]
			*n* = 124	*n* = 62 +TJ-41	*n* = 62 Chemo- and radio	Improves chemosensitivity and immunity.	The 4th people’s Hospital of Shenyang, China	[83]
	Gastric cancer Phase II/III	Oral 7.5 g/day 4 + 2weeks	*n* = 113	*n* = 56 TJ41	*n* = 57 (S1) S-1 ***	Improves adverse effects of chemotherapy.	Kyoto University Japan UMIN000004701	[90]
	Gastric cancer	Oral	*n* = 50/90/60/90	*n* = 25/45/30/45 +TJ-41	*n* = 25/45/30/45 Chemo-	Improves adverse effects of chemotherapy.	Jingjiang/Ruzhou/Yanling/Taihe, China	[84,85,86,87]
	Colon cancer	Oral decoction 28 days	*n* = 52	*n* = 27 +TJ41	*n* = 25 Chemo-	Improves diarrhea and adverse effects of chemotherapy.	Nanjing University of Chinese Medicine, China	[89]
TJ-43	Cancer-related anorexia	Oral 3 g/Bid 4 weeks	*n* = 56/4 *n* = 40 (total *n* = 90)	*n* = 26 *n* = 20 + TJ-43	*n* = 26 *n* = 20 Chemotherapy or radiotherapy	Improves dyspepsia and anorexia.	Daejeon Korean Medicine Hospital of Daejeon University KCT0002847	[104,105]
	Advanced esophagus cancer	Oral 2.5 g/Tid 2 weeks	*n* = 19	*n* = 8	*n* = 10 DFP	Improves CINV.	Tokushima University Hospital, Japan	[113]
	Esophagus cancer	Oral 2.5 g/Tid 22–35 days	*n* = 18	*n* = 9	*n* = 9 DFP	Improves anorexia	Hiroshima University, Japan	[115]
	Relapsed gastric cancer	Oral 2.5 g/Tid 2 weeks	*n* = 10	*n* = 5 +TJ43	*n* = 5 S-1 +CDDP	Improves CINV.	Gunma University, Japan	[112]
	Lung cancer	Oral 2.5 g/Tid 21–28 days	*n* = 60	*n* = 30	*n* = 30 CDDP	Improves cisplatin-induced anorexia.	JAPIC CTI-142747 Takeda General Hospital, Fukushima, Japan	[114]
		Oral 2.5 g/Tid 2 weeks	*n* = 40	*n* = 20	*n* = 20 CDDP		UMIN000010748 Hiroshima University, Japan	[116,117]
		Oral 2.5 g/Tid 1 week	*n* = 91	*n* = 64	*n* = 27 CDDP	Improves cisplatin-induced appetite.	Mito Medical Center Mito, Japan	[109]
		Oral 84 days	*n* = 100	*n* = 50	*n* = 50	Improves CINV, appetite, and fatigue.	Jiading Hospital, Shanghai, China	[122]
	Colon cancer	Oral 6 months	*n* = 70	*n* = 36	*n* = 34 5-FU	Improves CINV, diarrhea, and fatigue.	Jiading Hospital, Shanghai, China	[118]
		Oral 186 days	*n* = 70	*n* = 39	*n* = 39		Jiading Hospital, Shanghai, China	[121]
		Oral Bid 1 month	*n* = 60	*n* = 30	*n* = 30	Improves immunity and fatigue.	Jiangnan University Affiliated Hospital Jiangsu, China	[120]
	Gastric cancer	Oral 42 days	*n* = 64	*n* = 32	*n* = 32	Improves CINV, immunity, and fatigue.	Chuzhou Hospital Jiangsu, China	[119]
TJ-43	Cervical/corpus cancer	Oral 2.5 g/Tid 13 days	*n* = 40	*n* = 19	*n* = 17 CDDP + paclitaxel	Improves CINV and anorexia.	UMIN000011227 Phase II, 4 institutions, Hokkaido, Japan	[111]
	Dyspepsia	Oral 2.5 g/Tid 8 weeks	*n* = 247	+ TJ-43 *n* = 125	Placebo *n* = 122	Improves dyspepsia, epigastric pain, and postprandial fullness.	UMIN Clinical Trials Registry, Number UMIN000003954 (Japan)	[102]
TJ-48	Cancer-related anorexia	Oral 3 g/TID 4 weeks	*n* = 40	TJ-48	Placebo	Improves appetite and survival.	NCT02468141 (Korea) HI12C1889 (Completed)	[126,127]
	Cancer-related fatigue	Oral 3 g/TID 21 days	*n* = 48	+ TJ-48	Placebo	Improves fatigue (breast cancer).	KCT0003442 (Korea)	[153]
	HCC	Oral 7.5 g/day 6 years	*n* = 48	*n* = 10 + TJ-84	*n* = 38	Improves the recurrence-free survival.	University of Yamanashi Hospital (Japan) U19-ES11391 R01-AA16285 R01-ES12686	[124]
	Cancer-related fatigue	Oral 3 g/TID 56 days	*n* = 48	+ TJ-48	Placebo	Improves fatigue (breast cancer received doxorubicin and cyclophosphamide treatment).	NCT02858856 (Korea)	[130]
	Cancer-related fatigue	Oral	*n* = 16	+ TJ-48	N.A.	Improves QOL score. (NSCL)	Japan	[154]
	Non-small cell lung cancer	Oral 2.5g/TID 14 days~2 months	*n* = 45	*n* = 23 Chemo + TJ48	*n* = 22 Chemo- only	Improves the progression-free survival. Prevents nutritional disorders. Increases physical fitness.	Akita Red Cross Hospital (approval no. H26-7)	[155]
TJ-48	Breast cancer	3–5 g/TID 21 days	*n* = 79	+TJ-48 *n* = 13	Chemotherapy *n* = 66	Alleviates hepatotoxicity after chemotherapy. Enhances immune functions.	TMUH-02-10-02 Taipei, Taiwan	[156]
	Pancreatic cancer	Oral 7 years	*n* = 1	A case report	N.A.	Prevents adverse effects.	Tohoku University (Institutional Review Board No. 18,910)	[157]
PHY 906	Colorectal cancer	Oral 1.2 g/Bid 1.8 g/Bid 2.4 g/Bid 4 weeks	*n* = 17	*n* = 5 CPT-11/5-FU/LV + PHY906	*n* = 12 PT-11/5-FU/LV + placebo	Enhances efficacy of chemotherapy, reduces toxicity and alleviated side effects such as diarrhea, abdominal cramps, and vomiting.	PHY906-2000-1 (US)(Completed) Yale Cancer Center, HIC0808004167	[134,139]
				CPT-11 + PHY906	Placebo		PHY906-2002-1 (US) PHY906-2002-1-T (US)	
	HCC	Oral 800 mg/Bid	*n* = 31 (Phase I/II)	PHY906-+ Cape ***	Cape ***	Purpose: to evaluate the safety and efficacy of PHY906 NCT04000737	PHY906-2007-1-T NCT00076609 (Completed)	[142]
	Liver cancer	Oral 800 mg/Bid	*n* = 125 (Phase I)	PHY906 +Sorafenib	Sorafenib	Enhances efficacy of chemotherapy, reduces toxicity and alleviated side effects.	NCT04000737 (2020.03 updated)	[132]
	Pancreatic cancer	Oral 800 mg/Bid	*n* = 24 (Phase I/II)	PHY906- +Cape ***	N.A.	Improves survival, enhances efficacy of chemotherapy, reduces toxicity and alleviates side effects.	Yale Cancer Center, NCT00076609 NCT00411762 HIC0512000905 (2015.03 completed)	[135,137,140]

* Chemotherapy includes docetaxel, and low-dose 5-FU and cisplatin (CDDP). ** Treated with aprepitant–dexamethasone. *** S-1(dose: 80 mg/m^2^/d): includes capecitabine and oxaliplatin (CapeOX); and epirubicin, oxaliplatin and oxaliplatin (EOX).

## 4. Traditional Chinese Medicine as an Adjuvant Treatment to Improve the Side Effects of Cancer Therapy

Here, we summarize the use of TCMs to reduce some of the complications caused by chemotherapy or radiotherapy (Figure 2).

### 4.1. Fatigue

Fatigue is the most common side effect of cancer chemotherapy, and even adequate rest cannot alleviate the fatigue [158]. TJ-41 alleviates chemotherapy-induced fatigue, which may be attributed to the activation of the immune system [79]). In addition, TJ-41 alleviates chronic fatigue through the inhibition of interferon gamma (INF-γ), IL-6 and IL-1β [80], as well as halt the occurrence of inflammation [159]. Collectively, TJ-48, as a formulation, can be considered as enhancing health and immunity. Therefore, the combination of TJ-48 and chemotherapy could improve chemotherapy-induced malnutrition and prognosis, as it induces an improvement in nutritional intake that could increase physical strength [160]. 

### 4.2. Pain

Chronic pain is often seen after chemotherapy/radiotherapy treatments. Pain may result from cancer metastasis, or the nerve damage caused by cancer treatment [161]. More than 55% of patients suffer from pain during cancer treatment [162]. A previous study shows the ability of licorice to relieve pain in terminal cancer patients [163], and this may be due to the anti-inflammatory and antioxidative effects of licorice [164]. In chemotherapy treatment, paclitaxel is one of the most widely used chemotherapeutic drugs for several cancers. However, its side effects include the induction of neuropathic pain, which causes tingling and burning sensations [165]. Nuclear factor erythroid-2-related factor 2 (Nrf2) is considered as a regulator of antioxidant defense, and so its activation could ameliorate paclitaxel-induced pain [166]. The licorice-derived compound ISL, as a potential Nrf2 inducer, could upregulate Nrf2 expression and its downstream genes [167]. Licorice’s pain-relief potential may be due to its Nrf2-dependent transactivation ability and, thus, its antioxidant effect.

### 4.3. Mucosal Irritation

During clinical cancer treatment, radiotherapy and chemotherapy rapidly destroy high proliferative cells, including the proliferating cancer cells and the dividing epithelial cells [168]. In head and neck cancer patients, mucositis is the most common side effect of cancer therapy. In a double-blinded clinical trial, head and neck cancer patients received glycyrrhiza aqueous extract from the first day of radiotherapy, and it is found that this reduces the grade of mucositis and mucosal irritation after intervention [39]. Due to its anti-inflammatory effect, glycyrrhiza could inhibit macrophage activation, and decrease prostaglandin E2 levels and the secretion of free radicals in macrophages [169]. Moreover, its antioxidant action is able to scavenge free radicals and decrease the reactive oxygen species [170].

### 4.4. GI Side Effect

During chemotherapy treatment, toxicity causes GI side effects, including vomiting, diarrhea, and nausea [171]. The discomfort level may differ according to the type of chemotherapy, the duration, and the tolerance of patients. The emetogenic reagents include capecitabine [137], cisplatin, doxorubicin, and carboplatin [172]. In an in vivo study, PHY906 shows an ability to inhibit the nuclear factor kappa-light-chain-enhancer of activated B (NFκB), cyclooxygenase-2 (COX2), and inducible nitric oxide synthase (iNOS) pathways in CPT-11-induced intestinal inflammation [136]. Chemotherapy/radiotherapy-induced GI side effects could be reduced through the amelioration of the inflammatory factor.

### 4.5. Anemia 

Anemia in cancer patients occurs during disease progression, or as a result of blood loss, malnutrition, bone marrow damage, and radiation treatment [173,174], which impair erythropoietin (EPO) production and shorten the half-life of red blood cells [175]. Patients with anemia may suffer from dizziness, edema, heart failure, or even severe cognitive dysfunction [176]. TJ-48, which is abundant in ginsenoside, paeoniflorin, eudesmol, and glycyrrhizic acid, can stimulate bone marrow cells, alleviating anemia and, thus, reducing the decrease in hemoglobin [177,178]. In chronic hepatitis C, TJ-48 affects T cell–related immunity by improving peripheral blood T-helper (Th)1 cells, highlighting that the potential effect of TJ-48 may be related to its immune regulatory response [179].

### 4.6. Anorexia–Cachexia

Anorexia–cachexia is defined as a syndrome related to loss of appetite, weight loss, and invulnerable weight loss or muscle loss, which mainly results from a decrease in energy intake [180]. Loss of appetite is related to taste change, and is one of the most common side effects of cancer treatment [181]. During either cancer treatment or cancer progression, the production of appetite-depressing factors is activated through resultant oxidative stress or cytokine secretion [182]. Cancer treatment induces cytotoxic damage, which causes a rapid decrease in the taste and smell receptors and in the secretion of saliva, which results in the sense of the flavor of food being affected [183,184]. Yashtimadhu (*Glycyrrhiza glabra*) effectively decreases chemotherapy/radiotherapy-induced oral mucositis [185]. Licorice is also reported to prevent dexamethasone-induced muscle loss [186], and could effectively decrease muscle degradation-related proteins, and muscle RING-finger protein-1 (MuRF1) and atrogin-1 protein expression, by relying on its anti-oxidative effects. 

## 5. Traditional Chinese Medicine as an Adjuvant in Cancer Therapy

Chemotherapy/radiotherapy complications could be alleviated by several Kampo prescriptions. Moreover, several traditional herbal medicines are found to exert an adjuvant, anti-proliferative effect in cancer therapy. In a randomized controlled trial, adding TJ-41 for 2 weeks [76] not only improves cancer-related fatigue, but also decreases cancer therapy fatigue. These improvements may have benefits in different types of cancer, including breast, stomach, colorectal, and lung cancer. 

In a placebo-controlled clinical trial, TJ-48 is shown to improve appetite and survival after 4 weeks of oral administration. PHY906 is used widely in the treatment of GI symptoms, and has additional anticancer properties, as shown in a clinical trial where a combination of PHY906 and capecitabine treatments was given to pancreatic and GI malignancy patients [137]. Results show that 800 mg BID of PHY906, combined with different dosages of chemotherapy capecitabine, for 14 days, results in a better tolerance of capecitabine treatments, without any discomfort. In a previous clinical trial, PHY906 combined with irinotecan and 5-FU/LV treatment, for the treatment of colorectal cancer, lowers the frequency of GI-related side effects [139] through its cytoprotective and antidiarrheal activity. Moreover, in an in vivo study, PHY906 enhances CPT-11’s antitumor activity by apoptosis induction [187]. In hepatocellular carcinoma clinical treatment, sorafenib, the only approved drug, has diarrhea as a serious side effect. Interestingly, the combination with PHY906 not only enhances the sorafenib-induced autophagy by increasing p-AMPKα and p-ULK1, but also alleviates diarrhea [132]. Similarly, licorice aqueous root extract, combined with radiotherapy, may prevent oral ulcers. In a double-blinded clinical trial, head and neck cancer patients received glycyrrhiza aqueous extract from the first day of radiotherapy, which reduces the grade of mucositis and mucosal irritation [39]. 

Taken together, licorice-containing Kampo not only has the ability to improve cancer treatment-related side effects, but also has potential benefits as a cancer adjuvant therapy.

## 6. Bioactive Components of Licorice

**Figure 3:**
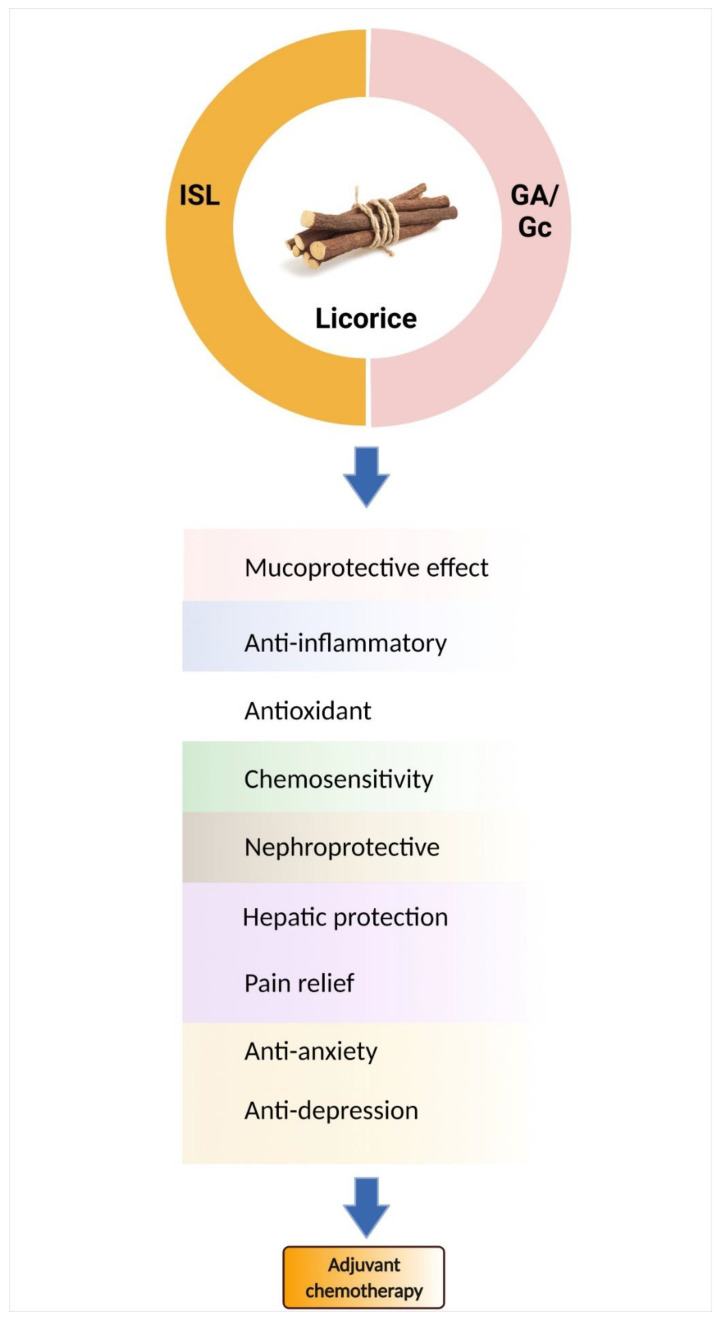
Licorice and its active components are candidates for chemo-combinations. *Glycyrrhizin* (Gc)/*glycyrrhetic* acid (GA) and isoliquiritigenin (ISL) mediate many mechanisms to improve chemotherapy-induced adverse effects.

Licorice root contains a variety of bioactive components, including alkaloids, polysaccharides, polyamines, triterpenes, phenolic acids, flavones, flavans, chalcones, flavonoids, and isoflavonoids. Among them, only a few can be characterized and isolated from licorice. In this review, only the components studied for chemoprevention are discussed (See Table 5), such as glycyrrhetic acid (GA) and chalcone-type derivative isoliquiritigenin (ISL) (Figure 3).

Glycyrrhizin demonstrates immunomodulatory actions in vitro, stimulating T lymphocytes for IL-2 production [188]. An anti-inflammatory effect is associated with glycyrrhizinic and glycyrrhetic acid, via an inhibition of corticosteroid metabolism and production [189]. To extend the corticosteroid effects, it is broadly classified into immunological and metabolic effects [190]. From a metabolic perspective, the active form of glycyrrhizin, glycyrrhetic acid, influences energy metabolism and fat distribution by mediating fatty acid oxidation–related genes [191,192]. To emphasize the role of antioxidants, pretreatment with glycyrrhizinic acid could decrease free radicals and increase the level of reduced glutathione (GSH) [193,194]. Licorice extract and glycyrrhizic acid could reduce ROS-mediating p53 activation, and promote p21 expression against cisplatin-induced nephrotoxicity in vitro [195]. In an animal model, glycyrrhizic acid (GA) and 18β-glycyrrhetinic acid (18βGA) are represented as chemoprotectants, through the modulation of the NF-κB and Nrf2 pathways to reduce cisplatin-induced nephrotoxicity [196]. Overall, glycyrrhizin has been widely studied for combination chemotherapies involving cisplatin, 5-Fluorouracil, radiation, doxorubicin, paclitaxel, etc. [196,197,198,199,200,201,202,203,204,205,206].

Isoliquiritigenin, one of the major bioactive compounds found in licorice, shares the same basic pharmacologic effects as *Glycyrrhiza* and exerts more biological activity, especially in its anti-tumor effects [10]. In a CT-26 murine colon animal model, ISL suppresses cisplatin-induced kidney/liver damage by mediating nitric oxide, lipid peroxidation, and GSH levels [207]. Based on the antioxidant properties of ISL, it shows a protective effect on cisplatin-induced toxicity, through regulating the oxidative ER stress hormesis. [208]. In addition, to target the anti-inflammatory effects, ISL also inhibits IL-6, IL-12, and TNF-α production [209]. Many studies suggest that licorice extract or licorice-derived active components benefit chemotherapy (Figure 3). Table 5 summarizes the licorice components associated with chemopreventive activities, mainly focusing on glycyrrhizin and ISL. However, some components of licorice present unwanted side effects; therefore, Kampo medicine is another option to improve chemotherapy-induced adverse effects.

**Table 5:** Licorice compounds, mechanisms of action and potential chemopreventions.

Compounds	Pharmacological Group	Chemotherapy	Therapeutic Actions/Mechanism	Ref
Glycyrrhizinic acid	Triterpenoid saponin	5-Fluorouracil	Mucoprotective effects, anti-inflammatory, and antioxidant (suppresses inflammatory mediators and oxidative stress via NF-κB and Nrf2 pathways) Enhances chemosensitivity (nitric oxide regulator)	[197,198]
		Cisplatin	Nephroprotective effect (inhibition of HMGB1)	[196]
		Cisplatin/radiation	Enhances chemosensitivity (1. decreases the expression of MRP2, MRP3, MRP4, and MRP5; 2. inhibition of HMGB1)	[199,200,201]
		Erlotinib/cisplatin	Enhances chemosensitivity (inhibition of progesterone receptor membrane component 1 (PGRMC1))	[202]
		Doxorubicin	Anti-inflammatory (decreasing phagocytosis of macrophage) Enhances chemosensitivity (mediates cell apoptosis via Bax/Bcl-2 ratio and caspase-3 activity) Cardioprotective (inhibition of HMGB1via HMGB1-dependent Akt/mTOR downregulating phospho-Akt, phospho-mTOR, p62, and LC3 II)	[204,205,210]
		Paclitaxel	Enhances chemosensitivity (via HMGB1/c-Myc inhibition)	[203,211]
			Anti-inflammatory (inhibition of NF-κB activation and IL-6 production)	[206]
		N.A.	Anti-anxiety and anti-depression (inhibition of HMGB1)	[212,213]
Glycyrrhizin		Cyclosporine (CsA)	Combined glycyrrhizin can reduce CsA-related liver injury, and attenuation of the severity of nausea and other adverse events	[214]
Isoliquiritigenin	Trans-chalcone (flavonoid)	Cisplatin	Antioxidant effects, and enhances chemosensitivity (enhances ER stress and oxidative stress) Enhances chemosensitivity (via HO-1 and GRP78/ABCG2)	[208,215,216,217]
			Nephro and hepatic protection (increases nitric oxide and tissue lipid peroxidation levels, and depletes GSH levels). Anti-inflammatory (inhibition of FPR2 in macrophage)	[207,218]
		5-Fluorouracil	Enhances chemosensitivity (induces p62/SQSTM1 by reducing caspase-8 activation)	[219]
			Immuno-protector (activation of macrophages and lymphocytes)	[220]
		Doxorubicin	Antioxidant effect, hepatic protection (via SIRT1/Nrf2 pathway)	[221]
			Enhances chemosensitivity	[222,223]

## 7. Conclusions

Currently, the utility of TCM in alleviating the adverse reactions induced by radiotherapy and chemotherapy in cancer treatment is gaining increased attention worldwide. Increasingly, evidence demonstrates that licorice-containing TCM can reduce chemotherapy- and radiotherapy-induced side effects, such as fatigue, appetite, GI toxicity, anemia, and mucositis. Collectively, licorice-containing TCM can improve patients’ QoL and reduce mortality. In this review, we conducted a descriptive study focusing on the role of the bioactive constituents in licorice-containing herbs in reducing the adverse effects of chemotherapy and radiotherapy. It is hoped that this comprehensive review will serve as a cornerstone to encourage more scientists to evaluate and develop effective TCM prescriptions, in order to improve the side effects of chemotherapy and radiotherapy.
